# Supplementary material for: Metabolic Tolerance to Atmospheric Pressure of Two Freshwater Endemic Amphipods Mostly Inhabiting the Deep-Water Zone of the Ancient Lake Baikal
Source: Insects. 2022 Jun 24;13(7):578. doi: 10.3390/insects13070578 (PMC9325015; doi:10.3390/insects13070578)

Figure S1. Raw data by depths of sampling. Bars indicate medians and dots show individual measurements. r — Pearson's coefficient describing correlation between the biochemical parameters right after sampling and the depths of amphipod collection.

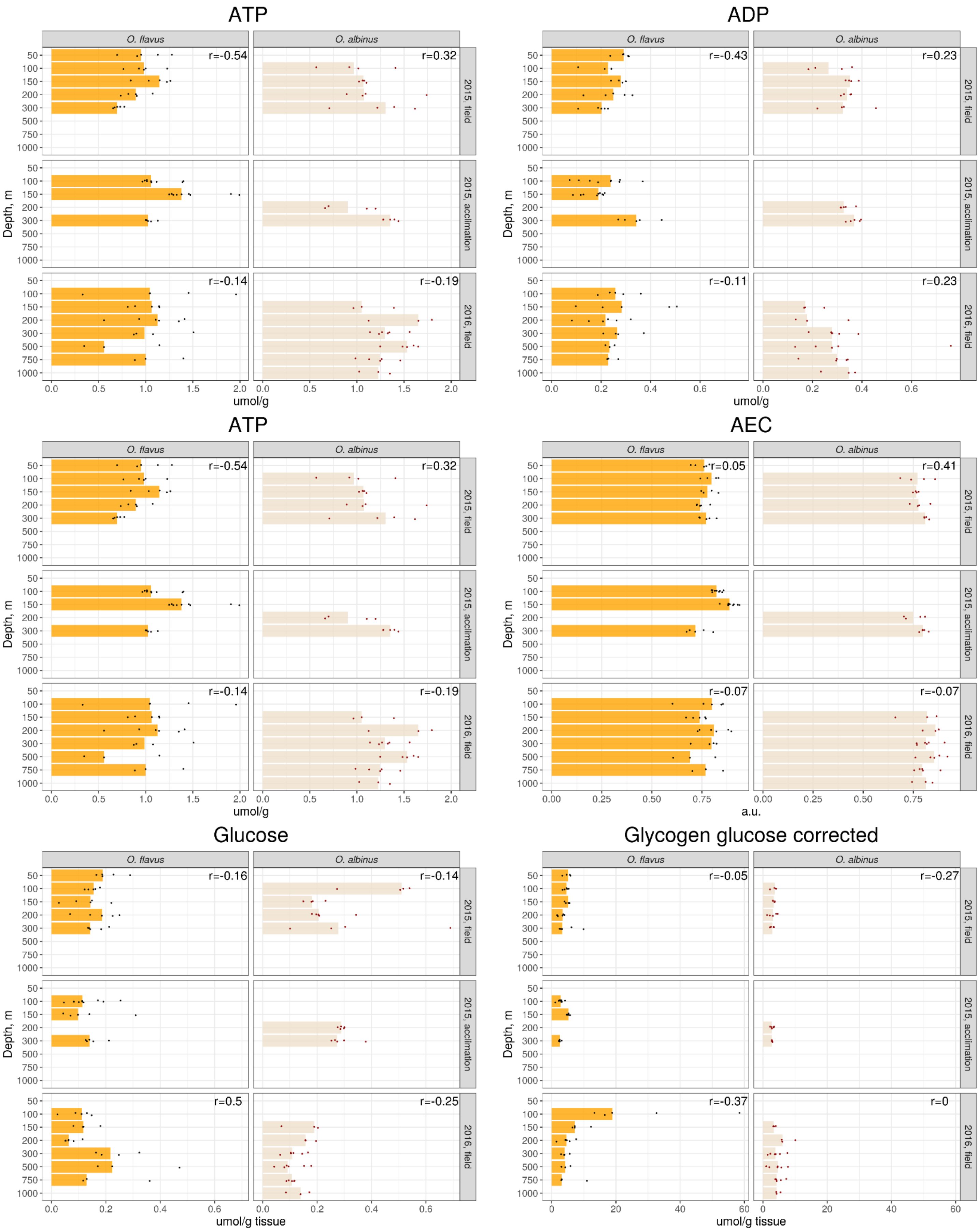

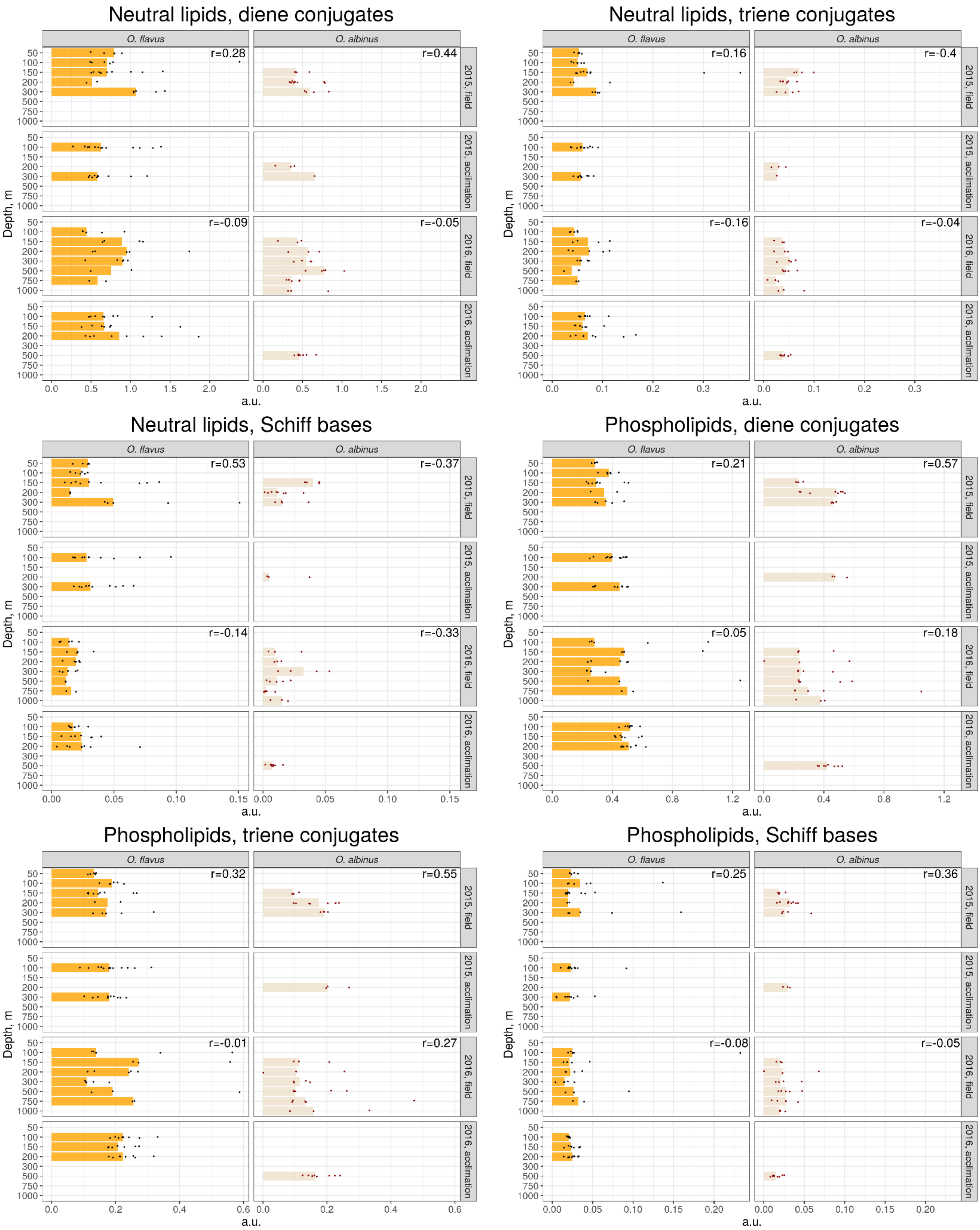
Neutral lipids, diene conjugates
Neutral lipids, triene conjugates
Neutral lipids, Schiff bases
Phospholipids, diene conjugates
Phospholipids, triene conjugates
Phospholipids, Schiff bases
O. flavus
O. albinus
2015, field
2015, acclimation
2016, field
2016, acclimation
Depth, m
a.u.
r=0.28
r=0.44
r=-0.09
r=-0.05
r=0.16
r=-0.4
r=-0.16
r=-0.04
r=0.53
r=-0.37
r=-0.14
r=-0.33
r=0.21
r=0.57
r=0.05
r=0.18
r=0.32
r=0.55
r=-0.01
r=0.27
r=0.25
r=0.36
r=-0.08
r=-0.05

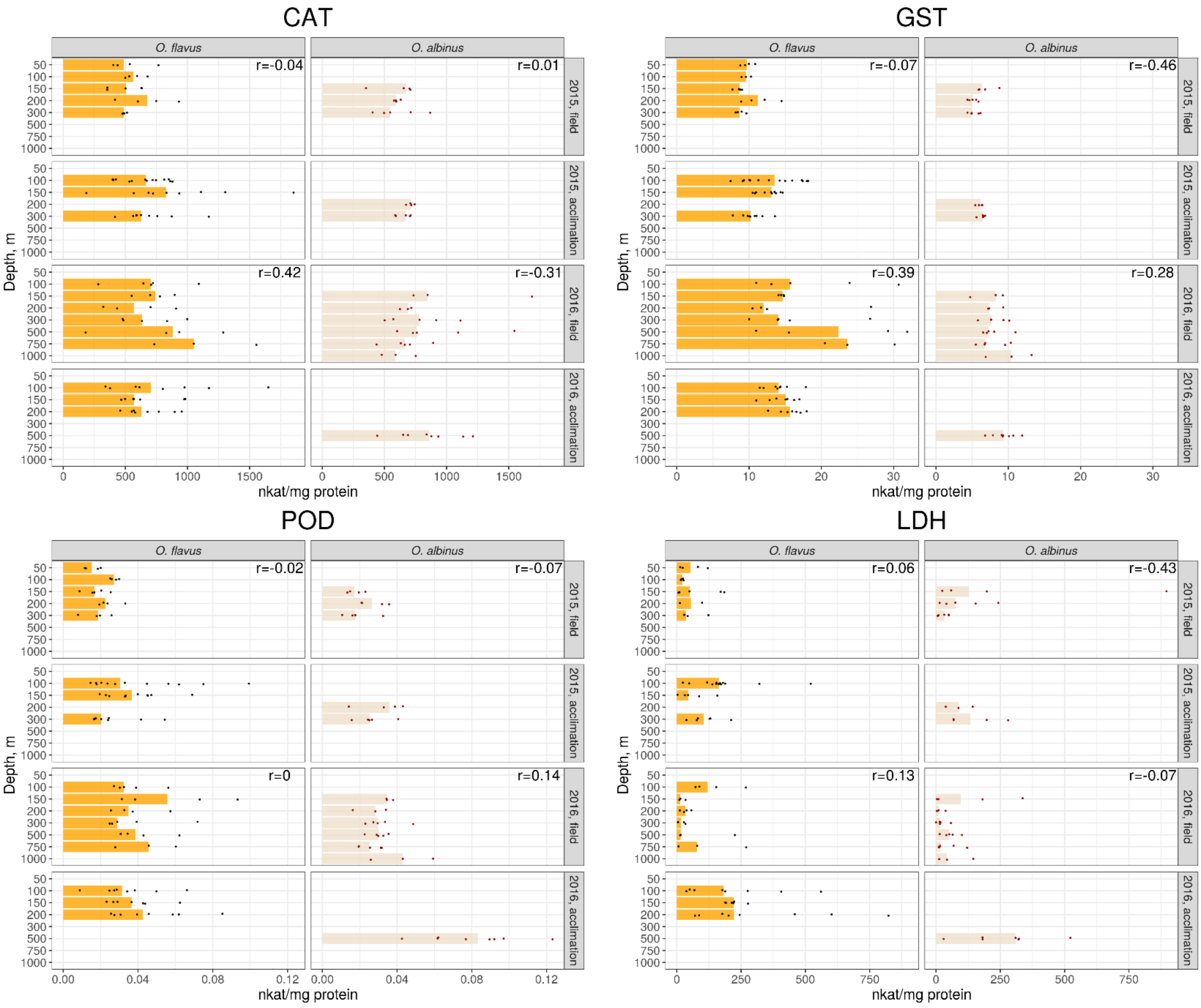
CAT
GST
POD
LDH
O. flavus
O. albinus
2015, field
2015, acclimation
2016, field
2016, acclimation
Depth, m
nkat/mg protein
r=-0.04
r=0.01
r=0.42
r=-0.31
r=-0.07
r=-0.46
r=0.39
r=0.28
r=-0.02
r=-0.07
r=0
r=0.14
r=0.06
r=-0.43
r=0.13
r=-0.07

Figure S2. Energy-related biochemical parameters in *O. flavus* and *O. albinus* fixed right after collection from deep-water traps in the range 100-300 m. Values are expressed as µmol g^{-1} of tissue for adenylates, glucose and glycogen (glucose-corrected) reserves and as nkat mg^{-1} for LDH activity. The numbers near the brackets indicate p-values for respective pairwise comparisons if the difference is statistically significant (Mann-Whitney U-test with Holm's correction for multiple comparisons).

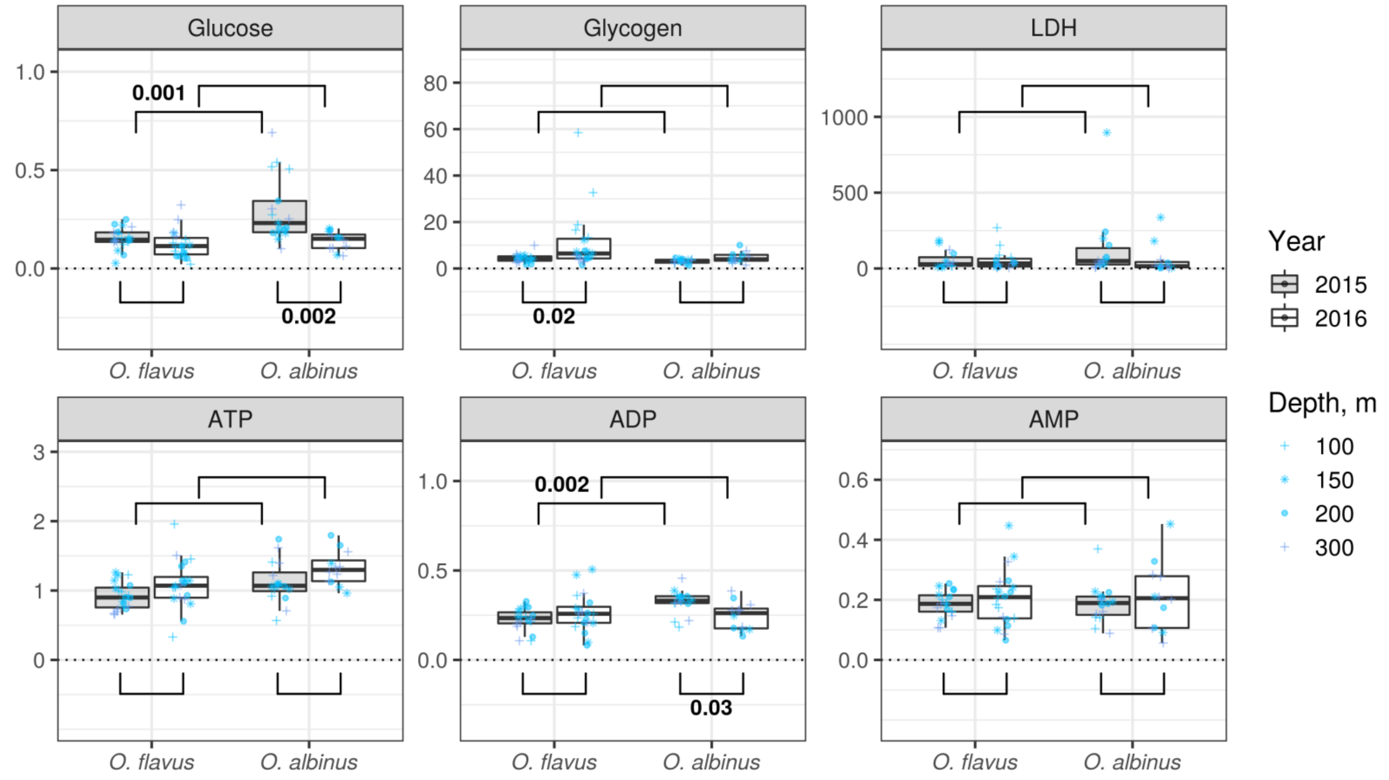

Figure S3. Biochemical parameters related to oxidative status in *O. flavus* and *O. albinus* fixed right after collection from deep-water traps in the range 100-300 m. Values are expressed as nkat mg^{-1} of protein for the enzyme activities (CAT, GST, POD) and in a.u. for lipid peroxidation products (DC, TC and SB). The numbers near the brackets indicate p-values for respective pairwise comparisons if the difference is statistically significant (Mann-Whitney U-test with Holm's correction for multiple comparisons).

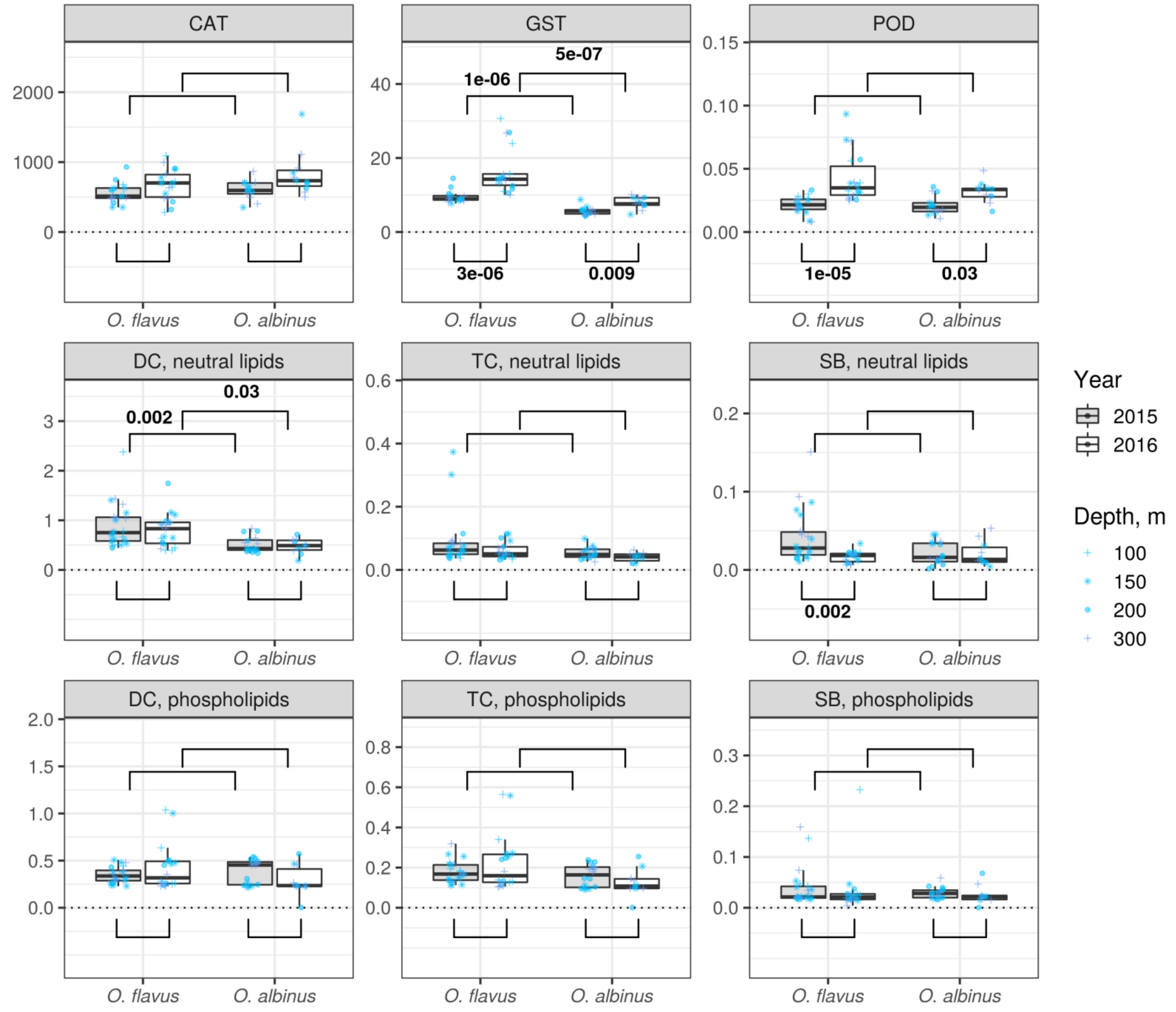

Supplement: Supplementary file 1 [file insects-13-00578-s001.zip › insects-1701879-supplementary/Supplementary Figures.pdf]
